# Immunogenicity of inactivated coronavirus disease 2019 vaccines in patients with chronic hepatitis B undergoing antiviral therapy

**DOI:** 10.3389/fmicb.2022.1056884

**Published:** 2022-11-30

**Authors:** Wen-Xin Wang, Rui Jia, Jin-Wen Song, Xiaoning Zhang, Shuang-Nan Zhou, Fu-Sheng Wang, Junliang Fu

**Affiliations:** ^1^Senior Department of Infectious Diseases, The Fifth Medical Center of Chinese People’s Liberation Army (PLA) General Hospital, National Clinical Research Center for Infectious Diseases, Beijing, China; ^2^Peking University 302 Clinical Medical School, Beijing, China; ^3^Department of Gastroenterology, The 985th Hospital of Joint Logistic Support Force of Chinese People’s Liberation Army (PLA), Taiyuan, China

**Keywords:** SARS-CoV-2, vaccination, hepatitis B virus, interferons, antiviral agents

## Abstract

**Objectives:**

To investigate the effect and its mechanisms of different antiviral agents on the immunogenicity of severe acute respiratory syndrome coronavirus 2 (SARS-CoV-2) vaccines in patients with chronic hepatitis B (CHB).

**Methods:**

A total of 125 patients with CHB receiving nucleos(t)ide analogs (NAs) monotherapy or combined with Peg-interferon-alpha (Peg-IFNα) therapy and 29 healthy controls (HCs) were enrolled. Adverse reactions (ADRs) and levels of neutralizing antibody (NAb), immunoglobulin G (IgG), immunoglobulin M (IgM), and peripheral cytokines post-vaccination were analyzed.

**Results:**

All ADRs were tolerable in CHB patients. Overall, no significant difference was observed in the antibody levels between patients and HCs after two doses of vaccination. An inverse correlation between NAb, IgG titers and the days after two doses was found in non-IFN group but not in IFN group. Correspondingly, peripheral interferon-γ levels were significantly higher in IFN group than in non-IFN group. After a booster dose, NAb and IgG antibodies were maintained at high levels in NA-treated patients.

**Conclusion:**

Peg-interferon-alpha-based therapy may be beneficial for maintaining the immunogenicity of SARS-CoV-2 vaccines in CHB patients, which may be related to the high levels of IFN-γ induced by Peg-IFNα therapy. A booster dose can effectively recall the robust and long-lasting immunogenicity of SARS-CoV-2 vaccines.

## Introduction

Since the outbreak of coronavirus disease 2019 (COVID-19), more than 630 million people have been infected with severe acute respiratory syndrome coronavirus 2 (SARS-CoV-2), and more than 6.6 million deaths were reported as of 2 November 2022 (Johns Hopkins University, 2022). Vaccines are economical and effective in controlling and preventing the spread of COVID-19, as well as reducing its disease severity (Hodgson et al., 2021). To date, more than 370 vaccines against SARS-CoV-2 are in clinical and pre-clinical development, including inactivated, mRNA, adenovirus-based, and recombinant protein vaccines, and approximately 12.8 billion doses have been administered (WHO, 2022).

Accumulated evidence has demonstrated that vaccines against SARS-CoV-2 have good safety and efficacy (Polack et al., 2020; Voysey et al., 2021), and can induce a humoral response against SARS-CoV-2 (Fix et al., 2021; Xia et al., 2021). The seroconversion rates of specific anti-receptor binding domain (RBD) immunoglobulin G (IgG) to inactivated SARS-CoV-2 vaccines were 70.37–86.67% (Bueno et al., 2022), while those to RBD-subunit vaccines were 72–76% and 93–97% after two and three vaccine doses, respectively (Yang et al., 2021). However, most clinical trials were conducted in healthy subjects; only a few participants with pre-existing liver diseases were enrolled (Cornberg et al., 2021; Fix et al., 2021; Marjot et al., 2021a). Several studies have demonstrated that people with specific comorbidities, including chronic liver disease (CLD), cardiac disease, and obesity, or those using certain medications, may be at greater risk of developing severe COVID-19 (Docherty et al., 2020; Fix et al., 2021). The European Association for the Study of the Liver, American Association for the Study of Liver Diseases, and Chinese medical association suggest that patients with chronic hepatitis B (CHB) having a stable condition and no contraindications should be vaccinated against SARS-CoV-2 (Cornberg et al., 2021; Fix et al., 2021; Chinese Society of Hepatology, 2021); however, safety and efficacy data on this population are still limited. Recently, three studies focused on CHB, CLD, and non-alcoholic fatty liver disease (NAFLD) and came to the same conclusion that inactivated SARS-CoV-2 vaccines are safe in these populations (Wang J. et al., 2021; Xiang et al., 2021; Ai et al., 2022). A study found that, compared with a healthy population, inactivated SARS-CoV-2 vaccines induced a lower immunological response in patients with CLD, which included 87.8% of patients with CHB (Ai et al., 2022). In contrast, another study found that patients with CHB undergoing nucleos(t)ide analogs (NAs) therapy had significantly higher neutralizing antibody (NAb) titers than those who were not (Xiang et al., 2021).

NAs and Peg-interferon-alpha (Peg-IFNα) are the two main anti-hepatitis B virus (anti-HBV) treatment regimens. Compared to NAs treatment, Peg-IFNα-based treatment strategies could achieve a higher rate of sustained hepatitis B e antigen and hepatitis B surface antigen loss/seroconversion (Hu et al., 2018; Liu et al., 2018, 2020), though low tolerability and a higher risk of adverse events exist (Yeh et al., 2019). Nowadays, approximately 90% and 10% of patients with CHB in China receive NAs and interferon, respectively (Shan et al., 2019). However, whether different antiviral agents have different effects on the safety and immunogenicity of SARS-CoV-2 vaccines requires further evaluation. In this study, we compared the adverse reaction (ADR) rates and antibody levels against SARS-CoV-2 in patients with CHB undergoing NAs monotherapy or Peg-IFNα-based treatment, and healthy controls (HCs).

## Materials and methods

### Patients and study design

A total of 125 patients with CHB and 29 HCs were enrolled in this cross-sectional study conducted at the Fifth Medical Center of the people’s liberation army (PLA) General Hospital (Beijing, China). None of the enrolled individuals had a history of COVID-19. All patients with CHB had normal liver function and received anti-HBV therapy during the vaccination period. Consecutive patients from May 7 to August 27, 2021, who received only one or two doses of vaccines were enrolled. Patients were divided into two groups: the IFN group receiving NAs combined with Peg-IFNα therapy (23 patients) and the non-IFN group receiving NAs monotherapy (74 patients). Consecutive patients who received a booster dose from January 21 to March 14, 2022, were also enrolled. Those patients received NAs monotherapy before enrollment. Patients with tumors or other hepatitis virus infections or liver cirrhosis were excluded from the study. CHB was diagnosed according to the guidelines for the prevention and treatment of chronic hepatitis B (2019 version) of the Chinese Medical Association (Wang and Duan, 2021). All patients and HCs received inactivated vaccines (0.5 ml/dose, Beijing Institute of Biological Products Co., Ltd., Beijing, China or Sinovac Biotech Co., Ltd., Beijing, China).

**FIGURE 1:**
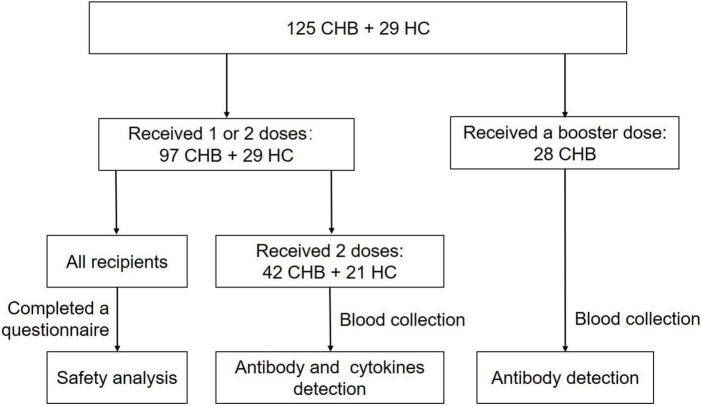
Flow chart of recipients enrolled. A total of 125 patients with chronic hepatitis B (CHB) and 29 healthy controls (HCs) were enrolled in this cross-sectional study. Safety analysis was performed on 97 patients and 29 HCs, who received one or two doses. Serum antibody and cytokines from 42 patients and 21 HCs were collected more than 14 days after two doses and analyzed. Another 28 nucleos(t)ide analog (NA)-monotherapy patients who received an additional booster dose (the interval from the second dose was 6–7 months) were examined for antibodies more than 14 days after three doses.

Safety analysis was performed on 126 recipients, including 79 patients and 26 HCs who received two doses with an interval of 3–7 weeks, and 18 patients with CHB and 3 HCs who received only one dose. Serum antibody and cytokines from 42 patients and 21 HCs were collected more than 14 days after vaccination and analyzed. Another 28 NA-monotherapy patients who received an additional booster dose (the interval from the second dose was 6–7 months) were examined for antibodies against SARS-CoV-2 (Table 1 and Figure 1). The research protocol was approved by the Ethics Committee of the Fifth Medical Center of the PLA General Hospital. All participants provided written informed consent. The study protocols conformed to the ethical guidelines of the latest version of the Declaration of Helsinki.

**TABLE 1:** Characteristics of subjects enrolled in this study.

	CHB	HC	*P*
Safety analysis	*N* = 97	*N* = 29	
Male/Female	73/24	12/17	0.001
Age (years)	40.0 (22, 62)	32.0 (22, 68)	0.000
Antibody and cytokines analysis after two doses	*N* = 42	*N* = 21	
Male/Female	29/13	8/13	0.019
Age (years)	39.5 (22, 60)	32.0 (22, 42)	0.004
Days after two doses	53.0 (14, 139)	89.0 (26, 150)	0.002
PSM for antibody analysis after two doses	*N* = 14	*N* = 14	
Male/Female	8/6	8/6	1
Age (years)	36.0 (23, 60)	37.0 (22, 42)	0.434
Days after two doses	72.5 (19, 133)	61.5 (26, 124)	0.550
Antibody analysis after three doses	*N* = 28	/	/
Male/Female	24/4	/	/
Age (years)	43.0 (27, 63)	/	/
Days after three doses	95.0 (14,133)	/	/

PSM, propensity score matching; CHB, chronic hepatitis B; HC, healthy control.

### Safety assessments

Safety assessments included the collection of local and systemic adverse reactions (ADRs) within 3 days after the first or second injection. Local ADRs included ache, redness, and swelling at the injection site, while systemic ADRs included fever, fatigue, nausea, headache, and muscular soreness. A questionnaire addressing the ADRs was completed by each participant who had received one or two injections.

### Antibody and cytokines detection

To detect NAb and IgG/immunoglobulin M (IgM) antibodies against the RBD of the SARS-CoV-2 spike protein, commercially available magnetic chemiluminescence enzyme immunoassay kits (Bioscience, Tianjin, China) were used according to the manufacturer’s instructions. A NAb value higher than 2.0 AU/ml was considered positive, while an IgG or IgM value higher than 1.0 S/CO was defined as positive. Cytokines [including interleukin-2 (IL-2), IL-4, IL-10, tumor necrosis factor-α (TNF-α), interferon-γ (IFN-γ), IL-17A] levels were determined using flow cytometry based on an Aimplex kit (Beijing QuantoBio, Beijing, China) following the manufacturer’s protocol.

### Statistical analysis

Categorical variables were analyzed using the x^2^ test or Fisher’s exact test. Continuous variables were expressed as median (minimum, maximum) and analyzed using the Mann–Whitney *U* test and Spearman correlation. Statistical analyses were conducted using SPSS 25.0, and statistical significance was set at *P* < 0.05. Considering the differences in demographic characteristics and days passed from the second dose of vaccination to blood drawing between patients with CHB and HCs, a 1:1 propensity score matching (PSM) of 0.2 caliper was conducted using R 4.1.1 to eliminate these differences (Table 1). Antibody levels were reanalyzed according to the PSM results.

## Results

### Adverse reactions after one or two doses

After two vaccine doses, 38 out of 97 (39.18%) patients with CHB and 10 out of 29 (34.48%) HCs experienced ADRs (*P* = 0.648) (Supplementary Table 1). A total of 61 ADR events occurred in 37 (38.14%) patients with CHB after the first dose, including 24 local ADR events in 23 patients, 31 systemic ADR events in 19 patients, and other ADRs, which were not included in the questionnaire, in six patients. In the HC group, 15 ADR events occurred in 10 recipients (34.48%) after the first dose, including six local ADR, seven systemic ADR, and two other ADRs. The most common local and systemic ADRs after the first dose were aching at the injection site and fatigue, respectively, in patients with CHB and HCs. In addition, no significant differences in the incidence and composition of ADRs were observed between patients with CHB and HCs. More than half of the patients with CHB (59.46%) and HCs (60.00%) who had ADRs after the first dose had just one type of ADR; only five patients and one HC had three or more types of ADRs. Most recipients experienced ADRs that had no or slight interference with regular work; only one patient with CHB required time off work. After the second dose, only 12 (15.19%) patients with CHB and four (15.38%) HCs had ADRs. All ADRs were mild or moderate and did not require medical intervention, and no recipient missed the second dose because of ADRs.

We further examined the occurrence of ADRs in patients treated with different anti-HBV drugs (Supplementary Table 2). After the first dose, the incidence of local ADRs in the IFN group (43.48%) was significantly higher than that in the non-IFN group (17.57%) (*P* = 0.011). In addition, the incidence of aches at the injection site in the IFN group (39.13%) was significantly higher than that in the non-IFN group (17.57%) (*P* = 0.031). Headache was the main systemic ADR in the IFN group, whereas fatigue was the main systemic ADR in the non-IFN group. No significant differences between the IFN and non-IFN groups were found in systemic ADRs, the number of types of ADR, the impact of ADRs on work after the first dose, and the incidence of ADRs after the second dose.

### No significant difference in antibody levels between patients with chronic hepatitis B and healthy controls after two doses of vaccines

**FIGURE 2:**
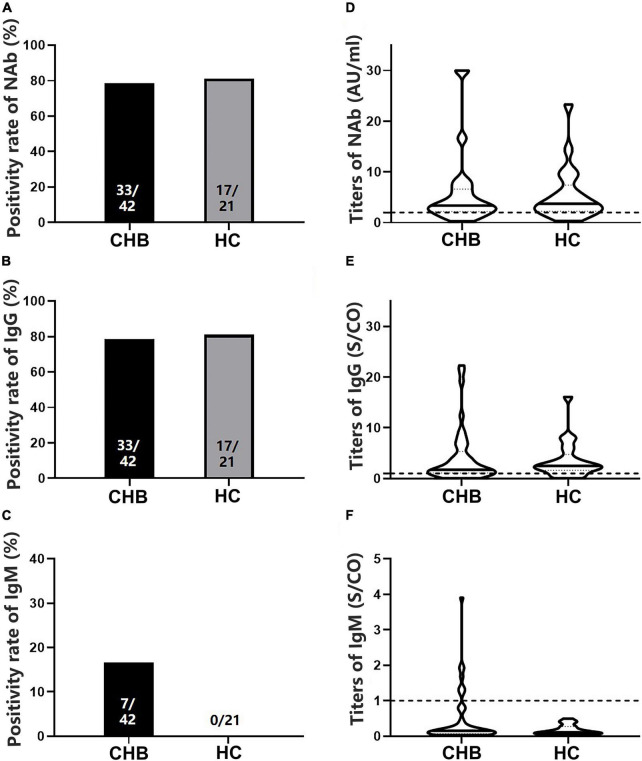
Comparison of the positivity rates and titers of antibodies against severe acute respiratory syndrome coronavirus 2 (SARS-CoV-2) between patients with CHB and HCs. **(A–C)** Comparison of NAb, immunoglobulin G (IgG), and immunoglobulin M (IgM) positivity rates between the two groups. **(D–F)** Comparison of NAb, IgG, and IgM antibody titers between the two groups; n1/n2 in bars, positive cases/all cases; dotted line, cutoff value; CHB, chronic hepatitis B; HCs, healthy controls; NAb, neutralizing antibody.

**FIGURE 3:**
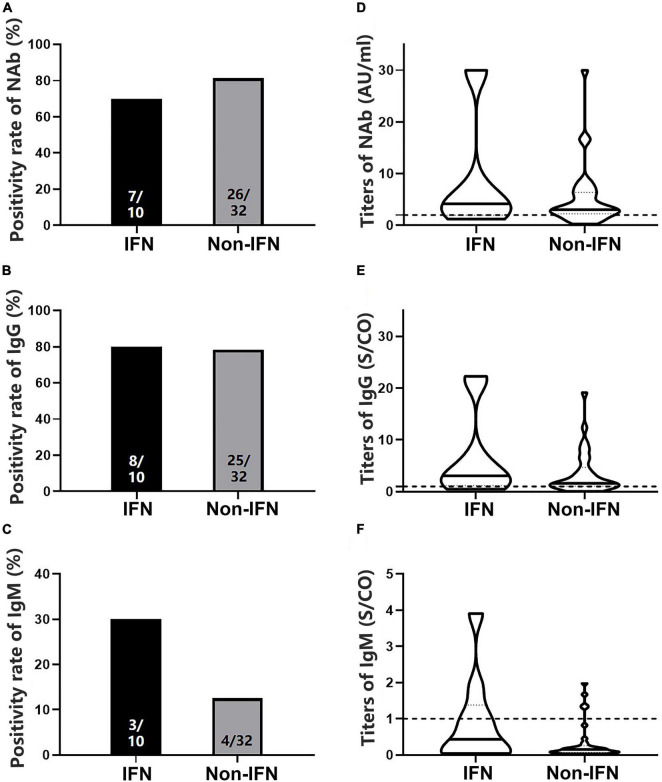
Comparison of the positivity rates and titers of antibodies against severe acute respiratory syndrome coronavirus 2 (SARS-CoV-2) between the interferon (IFN) and non-IFN groups among patients with CHB. **(A–C)** Comparison of NAb, immunoglobulin G (IgG), and immunoglobulin M (IgM) positivity rates between the IFN and non-IFN groups. **(D–F)** Comparison of NAb, IgG, and IgM antibody titers between the IFN and non-IFN groups. The IFN group, patients with CHB receiving nucleos(t)ide analogs (NAs) combined with Peg-interferon-alpha therapy; the non-IFN group, patients with CHB receiving NAs monotherapy; n1/n2 in bars, positive cases/all cases; dotted line, cut-off value; CHB, chronic hepatitis B; NAb, neutralizing antibody.

To analyze antibody levels after the second dose, blood samples were collected from 42 patients with CHB and 21 HCs (Table 1). No significant differences in NAb, IgG, or IgM positivity rates were observed between patients and HCs (78.57 vs. 80.95% for NAb, 78.57 vs. 80.95% for IgG, and 16.67 vs. 0.00% for IgM, *P* > 0.05) (Figures 2A–C), whether using Sinovac or Beijing Biologics SARS-CoV-2 vaccines (*P* = 0.662; *P* = 0.369). Similarly, no significant differences were observed in the antibody titers (*P* > 0.05) (Figures 2D–F). All HCs were negative for IgM, and only seven patients were IgM-positive (Figures 2C,F). Furthermore, no significant differences in NAb, IgG, and IgM positivity rates and titers were found between the IFN and non-IFN groups (*P* > 0.05) (Figure 3).

### The antibody levels declined significantly over time in patients with chronic hepatitis B

**FIGURE 4:**
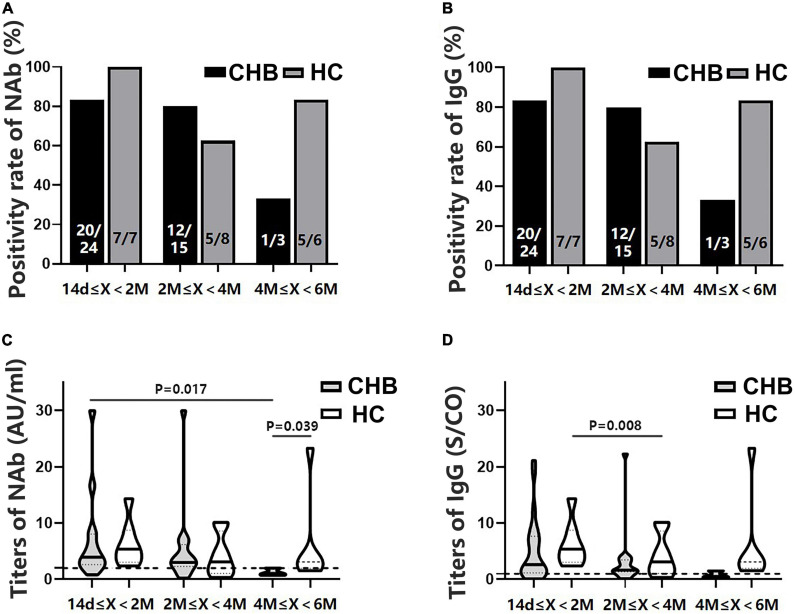
Comparison of the positivity rates and titers of antibodies against severe acute respiratory syndrome coronavirus 2 (SARS-CoV-2) between patients with CHB and HCs at 14 days–2 months, 2–4 months, and 4–6 months after the second vaccine dose. **(A,B)** Comparison of NAb and immunoglobulin G (IgG) positivity rates between the two groups at different time intervals. **(C,D)** Comparison of NAb and IgG antibody titers between the two groups at different time intervals; n1/n2 in bars, positive cases/all cases; X, days after the second dose; dotted line, cutoff value; CHB, chronic hepatitis B; HCs, healthy controls; NAb, neutralizing antibody.

All participants were divided into subgroups according to the time interval between antibody detection after the second dose: 14 days–2 months, 2–4 months, and 4–6 months. The positivity rates of NAb and IgG showed a marked downward trend in the patients, although the rates were not significantly different from those of the HCs (Figures 4A,B). Antibody titers showed a similar pattern (Figures 4C,D). The NAb antibody titers in the 4–6 months subgroup were significantly lower in patients than in HCs (*P* = 0.039). Moreover, these patients had lower NAb titers than those in the 14 days–2 months subgroup (*P* = 0.017) (Figure 4C).

**FIGURE 5:**
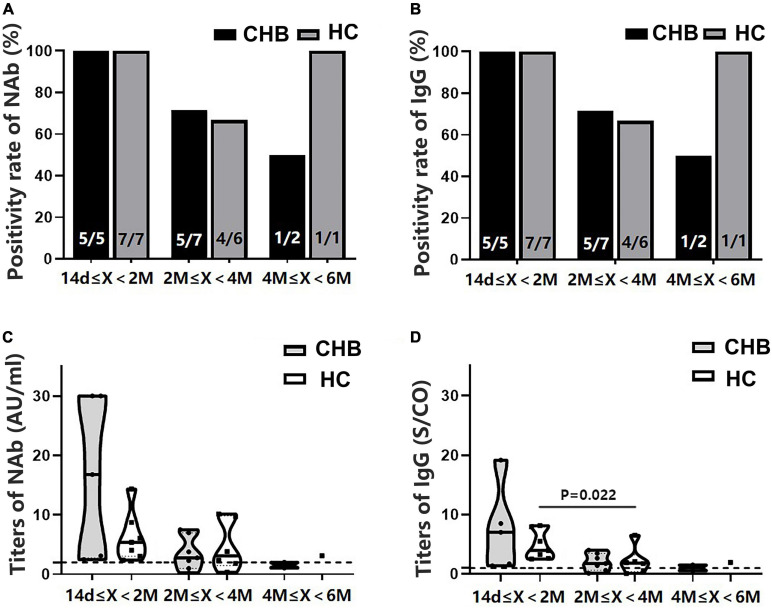
Comparison of the positivity rates and titers of antibodies after the second dose between patients with CHB and HCs after propensity score matching. **(A,B)** Comparison of the NAb and immunoglobulin G (IgG) positivity rates between the two groups at different time intervals. **(C,D)** Comparison of the NAb and IgG antibody titers between the two groups at different time intervals; n1/n2 in bars, positive cases/all cases; X, days after the second dose; dotted line, cutoff value; CHB, chronic hepatitis B; HCs, healthy controls; NAb, neutralizing antibody.

Since significant differences in sex, age, and time interval from vaccination to blood collection were observed between patients and HCs (Table 1), PSM was conducted. A total of 14 patients with CHB and 14 HCs were matched. Consistent with the results before PSM, no significant differences in NAb and IgG positivity rates and titers were found between the patients and HCs, and both antibody-positivity rates and titers showed an evident decreasing trend in the patients over time (Figure 5).

### Antibody levels inversely correlated with days after two doses of vaccination in the non-interferon group

**FIGURE 6:**
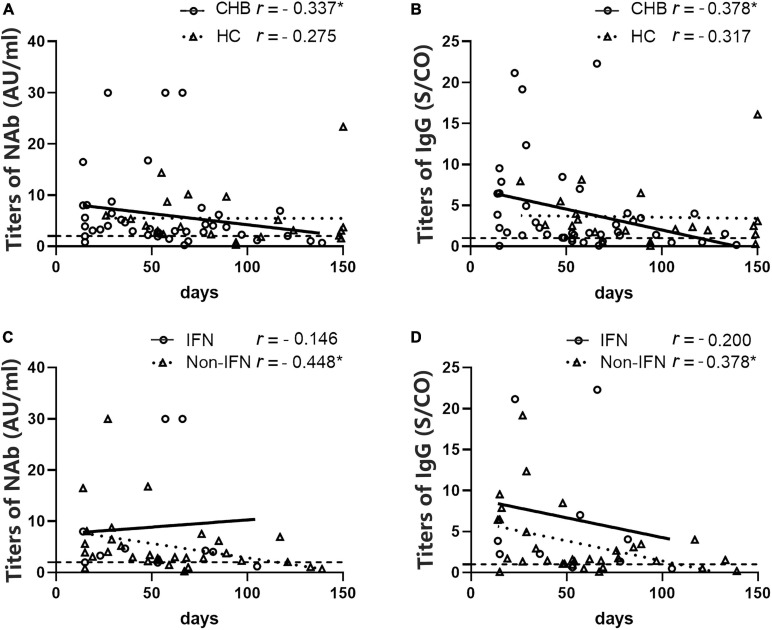
Analysis of the correlation between antibody titers and the number of days after the second vaccine dose in patients with CHB and HCs. **(A,B)** A negative correlation between NAb and immunoglobulin G (IgG) titers and the number of days after the second dose was observed in patients with CHB but not in HCs. **(C,D)** A negative correlation between NAb and IgG titers and the number of days after the second dose was observed in the non-IFN group of patients with CHB but not in the IFN group. *P < 0.05; the IFN group, patients with CHB receiving nucleos(t)ide analogs (NAs) combined with Peg-interferon-alpha therapy; the non-IFN group, patients with CHB receiving NAs monotherapy; n1/n2 in bars, positive cases/all cases; dotted line, cut-off value; CHB, chronic hepatitis B; HCs, healthy controls; NAb, neutralizing antibody.

Meanwhile, we found that the NAb and IgG antibody titers in the patients were inversely correlated with the days after the second dose (*r* = –0.337, *P* = 0.029; and *r* = –0.378, *P* = 0.014, respectively), whereas this relationship was not found in HCs (*r* = –0.275, *P* > 0.05; *r* = –0.317, *P* > 0.05, respectively) (Figures 6A,B). Interestingly, when patients were grouped according to the anti-HBV agents used, this correlation was only observed in the non-IFN group (*r* = –0.448, *P* = 0.010 for NAb; *r* = –0.378, *P* = 0.033 for IgG) (Figures 6C,D).

### Significantly increased peripheral interferon-γ levels in the interferon group

**FIGURE 7:**
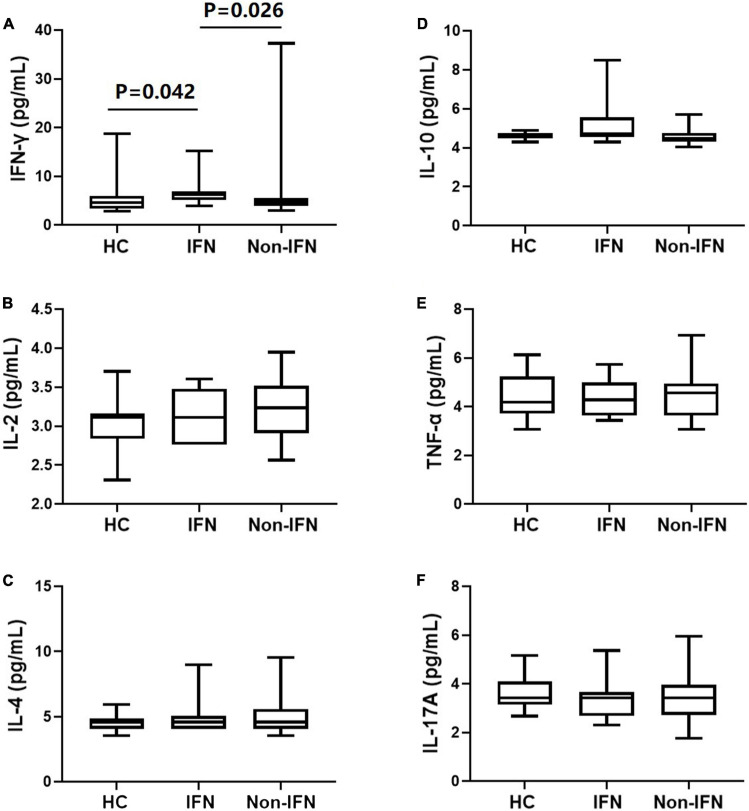
Comparison of serum cytokines in HCs, the IFN group, and the non-IFN group. **(A)** IFN-γ levels were significantly higher in the IFN group than in the non-IFN group and HCs. **(B–F)** No significant difference was observed in the peripheral levels of IL-2, IL-4, IL-10, TNF-α, and IL-17A among the three groups. The IFN group, patients with CHB receiving nucleos(t)ide analogs (NAs) combined with Peg-interferon-alpha therapy; the non-IFN group, patients with CHB receiving NAs monotherapy; IFN-γ, interferon-γ; IL, interleukin; TNF-α, tumor necrosis factor-α.

Cytokines levels in the peripheral blood were detected in the recipients after the second dose of the vaccine. We found that IFN-γ levels were significantly higher in the IFN group than in the non-IFN group (6.82 ± 3.15 vs. 6.21 ± 6.08 pg/ml, *P* = 0.026) and HCs (5.76 ± 3.89 pg/ml, *P* = 0.042) (Figure 7A). Furthermore, no significant difference in IFN-γ levels was observed between the non-IFN group and HCs (*P* = 0.506) (Figure 7A). There was no significant difference in the peripheral levels of other cytokines, including IL-2, IL-4, IL-10, TNF-α, and IL-17A, among the three groups (Figures 7B–F).

### High antibody levels were maintained after a booster dose in nucleos(t)ide analog-treated patients

**FIGURE 8:**
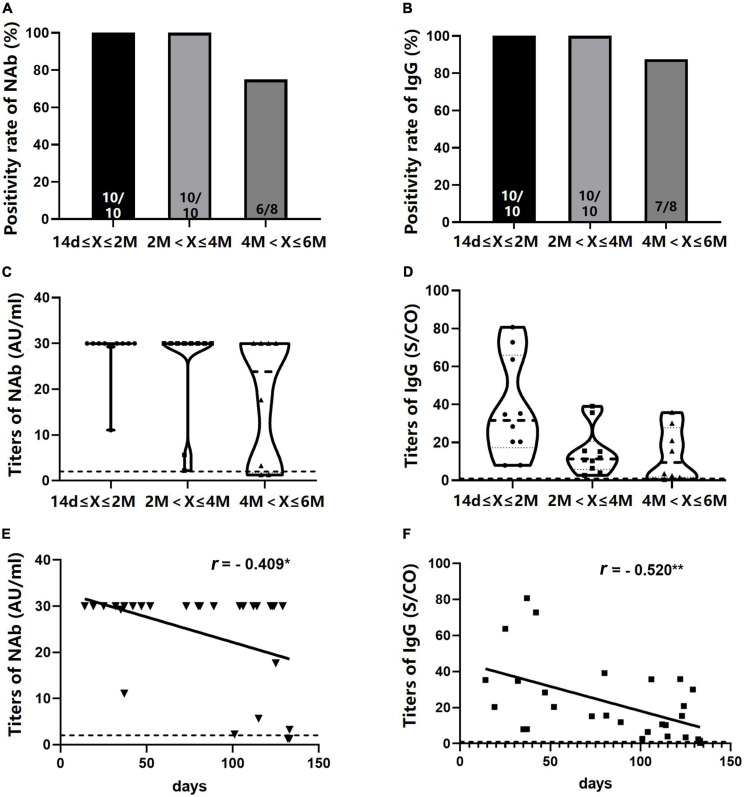
Seropositivity rates and titers of NAb and immunoglobulin G (IgG) in NA-treated patients after the booster dose. **(A,B)** NAb and IgG positivity rates at different time intervals after the booster dose. **(C,D)** NAb and IgG antibody titers at different time intervals after the booster dose. **(E,F)** A negative correlation was observed between NAb and IgG titers and the number of days after the booster dose. *P < 0.05; **P < 0.01; n1/n2 in bars, positive cases/all cases; dotted line, cut-off value; NAb, neutralizing antibody; NAs, nucleos(t)ide analogs. Symbols in C and D: ∙, antibody values in 14 days-2 months; □, antibody values in 2–4 months; ▲, antibody values in 4–6 months. ▼ in E: titers of NAb; □ in F, titers of IgG.

Neutralizing antibody and IgG levels were detected in 28 NA-treated patients who received a booster dose. The seropositivity rates of the two antibodies were 100% within 4 months after the third dose of the vaccine, dropping slightly within 4–6 months (75.0% for NAb and 87.5% for IgG) (Figures 8A,B). No significant difference was found in NAb and IgG titers across the three time intervals (14 days–2 months, 2–4 months, and 4–6 months) (Figures 8C,D); however, IgG titers showed an observable downward trend (Figure 8D). As expected, antibody titers were negatively correlated with the number of days after the third vaccination (NAb: *r* = –0.409, *P* = 0.031; IgG: *r* = –0.520, *P* = 0.005) (Figures 8E,F).

## Discussion

In the present study, we demonstrated that SARS-CoV-2 vaccines have acceptable safety in patients with CHB undergoing different antiviral therapies. The seropositivity rates of antibodies against SARS-CoV-2 vaccines were comparable between patients with CHB and HCs after two doses of the vaccine. However, both antibody seropositivity rates and titers declined noticeably over time in patients receiving NAs monotherapy than those receiving Peg-IFNα-based therapy.

An underlying disease is a risk factor for severe illness and death during SARS-CoV-2 infection. Several studies have shown that CLD is significantly associated with more severe COVID-19 and overall mortality (Kovalic et al., 2020; Kim et al., 2021). Preventive vaccination is one of the most effective ways to deal with the spread of the COVID-19. SARS-CoV-2 vaccines have been making unprecedented progress and have shown excellent safety profiles and efficacy in preventing symptomatic COVID-19 in healthy people (Marjot et al., 2021b). Although some studies have reported SARS-CoV-2 vaccination in patients with CHB, CLD, and NAFLD (Wang J. et al., 2021; Xiang et al., 2021; Ai et al., 2022), the safety and immunogenicity data on patients with CHB, especially those undergoing different anti-HBV drugs, is still insufficient.

The type and incidence of ADRs, predominantly occurring after the first dose, were similar between patients with CHB and HCs. All ADRs were self-limiting, tolerable, and quickly resolved. Our study did not observe any severe systematic ADR that required medical intervention. Further stratification analysis indicated that the incidence of ache at the injection site after the first dose was significantly higher in patients receiving Peg-IFNα-based therapy than in those receiving NAs monotherapy. This may be related to the short interval between Peg-IFNα injection and vaccination.

Recently, a clinical trial found higher levels of humoral immune responses in young and female recipients (Tanriover et al., 2021). Xiang et al. (2021) also reported that younger and female patients with CHB had higher anti-S-RBD IgG and NAb positive rates. A multinational, observer-blinded trial found similar vaccine efficacy across different subgroups defined by age (Polack et al., 2020). A higher proportion of male and older individuals in patients than in HCs were noted in our study, but no significant difference was found in IgG or NAb-positivity rates and titers between patients and HCs. Moreover, we also found no differences in antibody positivity rates and titers between patients of different ages and HCs (data not shown), suggesting that patients undergoing antiviral treatment can achieve the same level of vaccine protection as healthy people. This is consistent with the results reported by He et al. (2022) that seropositive rates of antibodies in CHB patients and HCs were similar at 1–3 months after vaccination. Xiang et al. (2021) observed that NA-treated patients with CHB can produce higher antibody levels than untreated patients. Ai et al. (2022) found no statistical difference in antibody seropositivity rate between patients undergoing antiviral therapy and those who did not. The inconsistent results may be due to the differences in sample size, liver function, and cirrhosis proportion of patients enrolled in the studies.

Specific antibody titers were correlated with protection against SARS-CoV-2 in animal models (Chandrashekar et al., 2020; Corbett et al., 2020). Clinical trials also revealed that vaccines with high levels of both binding and neutralizing antibodies were at low risk of severe illness and death (Anderson et al., 2020). Therefore, high levels of antibody titers against SARS-CoV-2 are associated with sufficient immune protective effects. Our data showed that seropositivity rates and titers of antibodies in patients with CHB declined more obviously 4–6 months after the second dose compared with HCs, indicating that the immune protection of the vaccine faded soon. A clinical trial demonstrated that administering a third vaccine dose 6–12 months after the second dose can boost immune response in healthy recipients (Wang K. et al., 2021). Our study firstly reported that an additional booster dose recalled robust and long-lasting antibodies against SARS-CoV-2 in NA-treated patients.

Interestingly, a negative correlation between the antibody titers and the days after vaccination only existed in patients receiving NAs monotherapy, but not in those receiving Peg-IFNα-based therapy. Compared with NAs, Peg-IFNα has an ability to stimulate humoral immunity and cellular immunity (Tang et al., 2018). In a clinical study, treatment with IFN-α2b limited the severity of COVID-19 (Zhou et al., 2020). In this study, we also detected higher levels of serum IFN-γ in patients receiving Peg-IFNα-based therapy. IFN-γ promotes macrophage activation and antigen presentation (Costela-Ruiz et al., 2020) and can enhance protective immunity when used as a vaccine adjuvant (Zhang et al., 2018). Furthermore, low levels of IFN-γ were associated with severe COVID-19 disease (Chen et al., 2020; Costela-Ruiz et al., 2020; Pedersen and Ho, 2020). Thus, we speculated that Peg-IFNα might help in maintaining high levels of antibodies against SARS-CoV-2 by promoting IFN-γ secretion.

Consistent with other findings (Banga et al., 2021), IgM antibodies against the RBD of the SARS-CoV-2 spike protein were not detected in most recipients in this study. This may be related to the fact that the vaccine mainly induces IgG antibody production (Banga et al., 2021). Another possible reason is the long interval between vaccination and blood withdrawal.

In conclusion, our study suggests that an additional booster dose with a shorter time interval is required for certain patients with CHB. However, our study has several limitations. First, this cross-sectional study had a small sample size and lacked dynamic observational data. Second, significant differences were found in the demographic characteristics and time of blood withdrawal between patients with CHB and HCs. Although PSM eliminated these differences, it led to a further reduction in cases. Third, neutralizing antibody detection cannot be equated to the virus neutralization test. Therefore, prospective longitudinal studies with larger sample sizes are needed to illustrate the characteristics of antibodies in patients with CHB and reveal the underlying immune mechanisms.

## Data availability statement

The original data presented in this study are included in the article/Supplementary material, further inquiries can be directed to the corresponding authors.

## Ethics statement

The studies involving human participants were reviewed and approved by the Ethics Committee of The Fifth Medical Center of PLA General Hospital Ethics Committees. The patients/participants provided their written informed consent to participate in this study.

## Author contributions

JF: study concept and design. W-XW, RJ, XZ, and S-NZ: acquisition of data. W-XW, RJ, and JF: analysis and interpretation of data. JF, W-XW, RJ, and J-WS: drafting of the manuscript. JF and F-SW: administrative, technical, or material support, study supervision. All authors contributed to the study conception and design, read and approved the final manuscript, including the authorship list, and critical revision of the manuscript for important intellectual content.
