# Supplementary material for: Immunogenicity of inactivated coronavirus disease 2019 vaccines in patients with chronic hepatitis B undergoing antiviral therapy
Source: Front Microbiol. 2022 Nov 30;13:1056884. doi: 10.3389/fmicb.2022.1056884 (PMC9748573; doi:10.3389/fmicb.2022.1056884)
Supplement: Supplementary file 1 [file Data_Sheet_1.docx]

Supplementary Material

# Supplementary Tables

**Supplementary Table S1.** Adverse Reactions of Subjects Enrolled in This Study.

| Adverse Reactions (ADRs) | CHB (n=97) | | HC (n=29) | | Comparison of Incidences(*P*) |
| --- | --- | --- | --- | --- | --- |
|  | No.(n) | Percentage (%) | No.(n) | Percentage (%) |  |
|  | Recipients/Events | Incidences/Composition | Recipients/Events | Incidences/Composition |  |
| **Total ADRs** | 38/80 | 39.18/100 | 10/21 | 34.48/100 | 0.648 |
| ADRs after 1st dose | 37/61 | 38.14/100 | 10/15 | 34.48/100 | 0.721 |
| ADRs after 2nd dose ^a^ | 12/19 | 15.19/100 | 4/6 | 15.38/100 | 1.000 |
| **Local ADRs after 1st dose** | 23/24 | 23.71/39.34 | 6/6 | 20.69/40.00 | 0.734 |
| Ache | 22/22 | 22.68/36.07 | 6/6 | 20.69/40.00 | 0.821 |
| Redness | 1/1 | 1.03/1.64 | 0/0 | 0/0 | 1.000 |
| Swelling | 1/1 | 1.03/1.64 | 0/0 | 0/0 | 1.000 |
| **Systemic ADRs after 1st dose** | 19/31 | 19.59/50.82 | 6/7 | 20.69/46.67 | 0.896 |
| Fever | 4/4 | 4.12/6.56 | 1/1 | 3.45/6.67 | 1.000 |
| <38.5℃ | 3/3 | 3.09/4.92 | 1/1 | 3.45/6.67 |  |
| ≥38.5℃ | 1/1 | 1.03/1.64 | 0/0 | 0/0 |  |
| <24 hours | 2/2 | 2.06/3.28 | 1/1 | 3.45/6.67 |  |
| ≥24 hours | 2/2 | 2.06/3.28 | 0/0 | 0/0 |  |
| Fatigue | 15/15 | 15.46/24.59 | 5/5 | 17.24/33.33 | 1.000 |
| Nausea | 2/2 | 2.06/3.28 | 0/0 | 0/0 | 1.000 |
| Headache | 5/5 | 5.15/8.20 | 0/0 | 0/0 | 0.480 |
| Muscle soreness | 5/5 | 5.15/8.20 | 1/1 | 3.45/6.67 | 1.000 |
| **Other ADRs after 1st dose** | 6/6 | 6.19/9.84 | 2/2 | 6.90/13.33 | 1.000 |
| **Number of types of ADR after 1st dose** |  |  |  |  | 0.935 |
| One | 22/22 | 22.68/59.46 | 6/6 | 20.69/60.00 |  |
| Two | 10/20 | 10.31/27.03 | 3/6 | 10.34/30.00 |  |
| Three | 2/6 | 2.06/5.41 | 1/3 | 3.45/10.00 |  |
| Four | 2/8 | 2.06/5.41 | 0/0 | 0/0 |  |
| Five | 1/5 | 1.03/2.70 | 0/0 | 0/0 |  |
| **Impact of ADRs on work** |  |  |  |  | 1.000 |
| Not at all | 27/27 | 27.84/72.97 | 8/8 | 27.59/80.00 |  |
| Slightly | 9/9 | 9.28/24.32 | 2/2 | 6.90/20.00 |  |
| Need time off | 1/1 | 1.03/2.70 | 0/0 | 0/0 |  |

a, 18 patients with CHB and 3 HCs did not receive the 2nd dose as they did not reach the scheduled time. Other ADRs included feeling down, sleepy, diarrhea, throat pain, palpitation, dreaminess. CHB: chronic hepatitis B; HC, healthy control; ADRs, Adverse Reactions.

**Supplementary Table S2.** Adverse Reactions of Enrolled CHB Patients with Different Anti-HBV Therapy.

| Adverse Reactions (ADRs) | Non-IFN Group (n=74) | | IFN Group (n=23) | | Comparison of Incidences(P) |
| --- | --- | --- | --- | --- | --- |
|  | No.(n) | Percentage (%) | No.(n) | Percentage (%) |  |
|  | Recipients/Events | Incidences/Composition | Recipients/Events | Incidences/Composition |  |
| **Total ADRs** | 26/51 | 35.14/100 | 12/29 | 52.17/100 | 0.144 |
| ADRs after 1st dose | 25/39 | 33.78/100 | 12/22 | 52.17/100 | 0.113 |
| ADRs after 2nd dose ^a^ | 6/12 | 10.00/100 | 6/7 | 26.09/100 | 0.054 |
| **Local ADRs after 1st dose** | 13/14 | 17.57/35.90 | 10/10 | 43.48/45.45 | 0.011 |
| Ache | 13/13 | 17.57/33.33 | 9/9 | 39.13/40.91 | 0.031 |
| Redness | 1/1 | 1.35/2.56 | 0/0 | 0/0 | 1.000 |
| Swelling | 0/0 | 0/0 | 1/1 | 4.35/4.55 | 0.237 |
| **Systemic ADRs after 1st dose** | 14/20 | 18.92/51.28 | 5/11 | 21.74/50.00 | 1.000 |
| Fever | 2/2 | 2.70/5.13 | 2/2 | 8.70/9.09 | 0.238 |
| <38.5℃ | 2/2 | 2.70/5.13 | 1/1 | 4.35/4.55 |  |
| ≥38.5℃ | 0/0 | 0/0 | 1/1 | 4.35/4.55 |  |
| <24 hours | 2/2 | 2.70/5.13 | 0/0 | 0.00/0.00 |  |
| ≥24 hours | 0/0 | 0/0 | 2/2 | 8.70/9.09 |  |
| Fatigue | 13/13 | 17.57/33.33 | 2/2 | 8.70/9.09 | 0.485 |
| Nausea | 0/0 | 0/0 | 2/2 | 8.70/9.09 | 0.054 |
| Headache | 2/2 | 2.70/5.13 | 3/3 | 13.04/13.64 | 0.156 |
| Muscle soreness | 3/3 | 4.05/7.69 | 2/2 | 8.70/9.09 | 0.734 |
| **Other ADRs after 1st dose** | 5/5 | 6.76/12.82 | 1/1 | 4.35/4.55 | 1.000 |
| **Number of types of ADR after 1st dose** |  |  |  |  | 0.591 |
| One | 15/15 | 20.27/60.00 | 7/7 | 30.43/58.33 |  |
| Two | 7/14 | 9.46/28.00 | 3/6 | 13.04/25.00 |  |
| Three | 2/6 | 2.70/8.00 | 0/0 | 0.00/0.00 |  |
| Four | 1/4 | 1.35/4.00 | 1/4 | 4.35/8.33 |  |
| Five | 0/0 | 0/0 | 1/5 | 4.35/8.33 |  |
| **Impact of ADRs on work** |  |  |  |  | 0.372 |
| Not at all | 18/18 | 24.32/72.00 | 9/9 | 39.13/75.00 |  |
| Slightly | 7/7 | 9.46/28.00 | 2/2 | 8.70/16.67 |  |
| Need time off | 0/0 | 0/0 | 1/1 | 4.35/8.33 |  |

a, 14 in the non-IFN group and 4 in the IFN group did not receive 2nd dose as they did not reach the scheduled time. Other ADRs included feeling down, sleepy, diarrhea, throat pain. The IFN group: CHB patients receiving nucleos(t)ide analogs combined with Peg-interferon-alpha therapy; the non-IFN group: CHB patients receiving nucleos(t)ide analogs monotherapy; CHB: chronic hepatitis B; anti-HBV, anti-hepatitis B virus; ADRs, Adverse Reactions.
